# A Hybrid Methodology to Minimize Freshwater Consumption during Shrimp Shell Waste Valorization Combining Multi-Contaminant Pinch Analysis and Superstructure Optimization

**DOI:** 10.3390/polym13111887

**Published:** 2021-06-06

**Authors:** Viviana Quintero, Arturo Gonzalez-Quiroga, Angel Darío Gonzalez-Delgado

**Affiliations:** 1 Nanomaterials and Computer Aided Process Engineering Research Group (NIPAC), Chemical Engineering Department, University of Cartagena, Avenida del Consulado St. 30, Cartagena de Indias 130015, Colombia; vquinterod@unicartagena.edu.co; 2 UREMA Research Unit, Mechanical Engineering Department, Universidad del Norte, Barranquilla 25138, Colombia; arturoq@uninorte.edu.co

**Keywords:** shrimp exoskeleton, water network, chitosan, pinch analysis multiple contaminants, mathematical program

## Abstract

The conservation and proper management of natural resources constitute one of the main objectives of the 2030 Agenda for Sustainable Development designed by the Member States of the United Nations. In this work, a hybrid strategy based on process integration is proposed to minimize freshwater consumption while reusing wastewater. As a novelty, the strategy included a heuristic approach for identifying the minimum consumption of freshwater with a preliminary design of the water network, considering the concept of reuse and multiple pollutants. Then, mathematical programming techniques were applied to evaluate the possibilities of regeneration of the source streams through the inclusion of intercept units and establish the optimal design of the network. This strategy was used in the shrimp shell waste process to obtain chitosan, where a minimum freshwater consumption of 277 t/h was identified, with a reuse strategy and an optimal value of US $5.5 million for the design of the water network.

## 1. Introduction

Global competitiveness policies and environmental regulations have motivated industries to direct their design strategies towards the valorization of intermediate streams called waste, allowing them to diversify supply and reduce environmental impacts. [1]. In the shrimp industries, around 48% of the shrimp is discarded as waste, which includes the shell and the head [2]. However, the presence of high added value compounds such as pigments, chitin, and lipids in this material has allowed the design of different valorization strategies widely exposed in the literature [3]. One of them is obtaining polymer chitosan composed of β- (1–4) D-glucosamine units with physicochemical properties such as biodegradability, biocompatibility, bioactivity, and low toxicity. For that reason, this compound has been investigated in different fields, for example, antimicrobial activity, wastewater treatment, food industries, nanoparticles, and biopolymers, among others [4,5]. Chitosan is produced by enzymatic or chemical deacetylation of chitin; this last route is characterized by high freshwater consumption and availability of intermediate streams. In this paper, a strategy to minimize the consumption of fresh water and disposal of waste streams is presented [6]. The applied strategy is based on the synthesis methodology that involves two fundamental activities: water network design (WN) and the superstructure model solution. The water network design (WN) is based on the concept of the superstructure, embedding all possible process units and all the connections among resources, interceptors, process units, and wastewater treatment [7].

Sources are streams from process units that have water available to be recycled and reused [8]. The stream sources are purified partially by given regeneration units with established performance indexes before. Different technologies used for this purpose are presented with known removal ratios and design parameters in the literature. However, prior identification of the principal pollutants is essential, because this influences the water network’s economic feasibility [9].

On the other hand, sinks are process units that require water but present restrictions in terms of flow and stream composition. An external freshwater source is included to supply the unit sink’s flow rate requirement. Finally, the wastewater treatment units treat the streams not received by the sink [10]. The water network main designs are identified in the literature: water-using networks (WUNs) and total water network (TWN). The WUNs use processes and treatment and/or regeneration units to increase the reuse potential of available water; however, wastewater treatment is considered a discharge unit that is not included in the configuration. In TWN, the water-using units, regeneration units, and treatment units are combined into a single network, with an environmental constriction on the concentration of streams leaving the TWN [10].

The activity related to the superstructure model solution uses pinch methodology and mathematical programming techniques [11].

### 1.1. Pinch Methodology

The contributions related to the pinch methodology have been directed mainly to the identification of the minimum consumption of fresh water and wastewater disposal, taking into account reuse, recycling, and regeneration strategies, with single or multiple contaminants, through graphical and algebraic approximations and their combinations [12]. Foo [13] presented a review of existing strategies for water network synthesis using the pinch methodology. The techniques were classified into fixed load and fixed flow for flow rate targeting techniques, water reuse networks, and wastewater treatment. He identified the most advanced techniques as limiting compositive curves, material recovery pinch diagrams, and water cascade analyses, which correspond to fixed flow and single contaminant.

Otherwise, Klems [14] shows an analysis of the progress published on the pinch methodology in areas such as heat exchangers, exergy, and water network synthesis. The author included the compilation of existing techniques and guidance for future developments. Regarding the water network synthesis, strategies are identified that include the combination of the pinch of water with the water footprint and sequential methods that involve the objective flow of resources and the synthesis of the network from mathematical methods; however, these strategies are limited to a single pollutant [15].

### 1.2. Mathematical Programming

From a mathematical programming viewpoint, deterministic methods have been widely used in the water network’s optimal design, addressing it as MINLP problems (mixed integer nonlinear programming) [16] and their combinations of linearization and relaxation, which lead to MILP (mixed integer linear programming) [17], NLP (nonlinear programming), and meta-heuristic algorithms [18]. These methods involve global and component mass balances such as constraints and single or multiobjective functions addressed to maximizer or minimizer criteria [19].

In this context, the single objective provides a global optimal solution directed to a criterion such as total annualized cost, minimum freshwater consumption/wastewater generation, or the minimum number of interconnections [20]. A multi-objective optimization framework is developed to optimize different objectives, especially those in conflict with different functions [21]. These involve several criteria (economic, technical, and environmental), providing a virtually infinite number of equally effective solutions (i.e., the Pareto front) that are trade-off solutions between objectives. According to his criteria, the best solution among the set of solutions must be identified by the analyst [22].

The present paper proposes a hybrid methodology, which seeks to integrate the heuristic and mathematical programming approach through a pinch analysis for multiple pollutants followed by mathematical programming synthesis. It allows a holistic design and obviates complex mathematical formulations for the solution of the proposed network. This strategy has been designed in four sequential steps. First, the base scheme’s modeling and simulation were directed to obtain specific information about current availability (source) and restrictions on sink units (flows and concentrations of pollutants). Second, the calculation of the minimum consumption of fresh water in the process was carried out applying the methodology proposed by Chin et al. [23], where the concept of “contaminant cascades” was developed.

This indicates that the prioritization of sources and sinks was carried out according to the contaminant concentration in ascending order. Third, the water network superstructure construction was carried out, considering reuse and regeneration strategies to analyze possible current interactions and intercepted units’ viability. Finally, a model based on the built superstructure involves global mass balances and pollutants in each process unit. The objective function minimizes the total network cost consisting of freshwater, the investment cost of treatment units, and the operating cost for the treatment, taking freshwater as a restriction. The model was solved in Gams.

## 2. Materials and Methods

**Figure 1:**
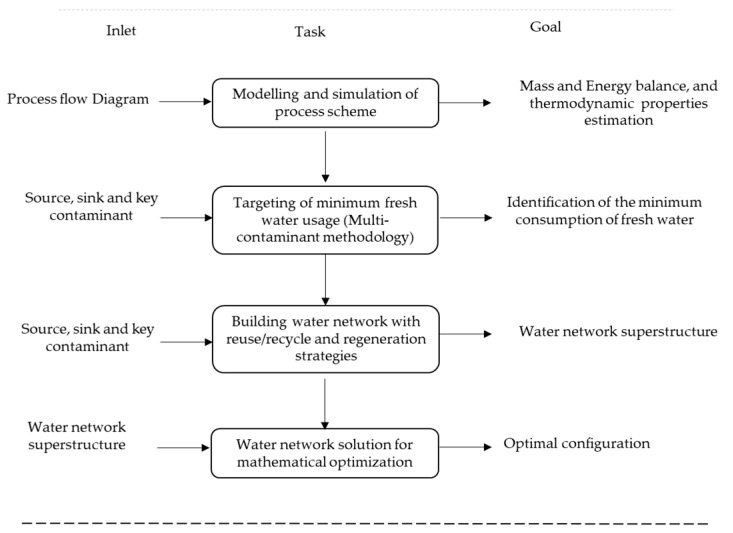
Methodological design.

The approach proposed here is divided into four sequential stages, as is shown in Figure 1: modeling and simulation of the process scheme, targeting of minimum freshwater usage (multi-contaminant methodology), building the water network with reuse/recycle and regeneration strategies, and the water network solution for mathematical optimization.

### 2.1. Modeling and Simulation of Process Scheme

Two sequential tasks were developed. The first one was related to the process’s conceptual model construction, which involves the study object definition and its surroundings. Relevant data, such as calculation base, operating conditions, and yields, were collected. The second refers to modeling and simulation, where the conceptual model was described through mathematical expressions and programming environments, and the proposed model was resolved. It is common in chemical processes to use software such as Pro II, Aspen Plus, and ProSimPlus. In this paper, Aspen Plus version 10.4 was used for mass and energy balance and thermodynamic properties estimation [24].

### 2.2. Targeting of Minimum Freshwater Usage

The minimum consumption of freshwater was calculated following the methodology proposed by Chin et al. [23]. The sink process units d were first identified, where d ϵ D and $D=N_{sink}$, with the flow required $F_{SKd}$ and the maximum concentration of contaminant allowed $Z_{k,skd}$. The water stream source that can be recycled or reused to fulfill the sinks r ϵ R $R=N_{source}$ with available flow $FS_{\;r}$ and the pollutant concentration $C_{k,sr}$ Then, two sequential stages were carried out, a pre-targeting procedure in which the limiting contaminants for each sink and the possible sequence for the source’s use were determined, followed by the sinks’ location above and below the pinch point and their combination in each region’s single sink. The second stage was source allocation, where the minimum point of the freshwater requirement was determined.

### 2.3. Building the Water Network with Reuse/Recycle and Regeneration Strategies

**Figure 2:**
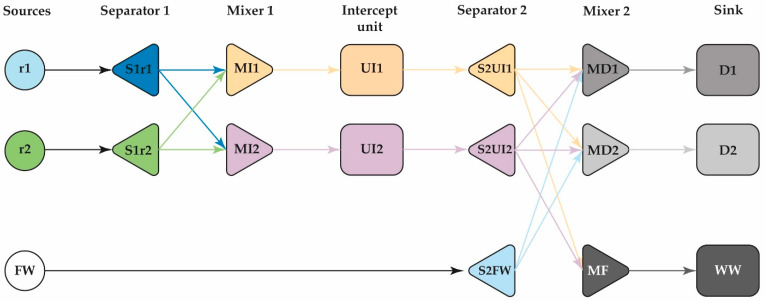
General superstructure design.

To further reduce the amount of freshwater required, a synthesis for the water network is proposed, as shown in Figure 2. Design assumptions are listed below:

The source streams have a flow $FS_{\;r}$ and a defined concentration $C_{k,sr}$ Like sink units, they have a flow $F_{SKd}$ and maximum allowed pollutant concentration $Z_{k,skd}$ The freshwater streamflow $F_{freshwater}$ is defined from the pinch analysis with a zero-pollutant concentration of pollutant. It includes a set of intercepted units $U_{t}$ with fixed recoveries $C_{k\;}^{out}=\beta_{tk}C_{k}^{in}$, where $\beta_{tk}$ is the recovery of contaminant *k* in $U_{t}$ Mixers were included in the superstructure to distribute the streams to each of the process units.to avoid non-linearities due to the balance of properties at the mixing point before each sink and unit intercept process. A fictitious unit called bypass was included within the intercepts to direct the currents with properties that are not intercepted, with effectiveness and cost equal to zero.

### 2.4. Water Network Solution for Mathematical Optimization

The water network solution was carried out, taking into account two aspects: the formulation of the mathematical model and its respective solution.

The mathematical model includes the formulation of the restrictions and the objective function. For the superstructure of the water network, the restrictions consist of mass balance equations for water. The contaminants for every unit are presented as follows and are based on what it proposes [25]. Each source process was segregated toward different interceptors. Equation (1) shows the general balance of the source in the separators.

(1) $$FSU_{r}^{out}=\underset{i\;\in IU}{\sum }FSI_{r,i}\;\forall r\;\in$$

The set of streams from the splitter process source unit is sent to mixer 1. The outlet stream from the mixer unit is directed to the intercept unit. The overall material balance for the mixer process unit is given by Equation (2), and the mass balance for each contaminant *j* by Equation (3).

(2) $$FIU_{i}^{inlet}=\underset{r\;\in SU}{\sum }FSI_{r,i}\;\vee i\;\in IU$$

(3) $$FIU_{i}^{inlet}*xIU_{i,k}^{inlet}=\underset{r\;\in UF}{\sum }FSI_{r,i}*xUS_{r,k}^{out}\;\forall \;i\;\in IU,\;\forall \;j$$

In the unit intercept *IU*, the inlet $FUI_{i}^{inlet}$ and outlet $FUI_{i}^{out}$ stream flows are equal. Equations (4) and (5) show an overall balance and for contaminant *k*, assumed to be a linear function in terms $R_{i,j}$ On the other hand, a fictitious unit is included when the property is not intercepted with effectiveness and cost equal to zero.

(4) $$FUI_{i}^{inlet}=FUI_{i}^{out}\;\forall i\;\in UI$$

(5) $$xUI_{i,j}^{out}=\beta_{UI\;i,k}*xUI_{i,k}^{inlet}\;\forall \;i\;\in UI,\;\forall \;k$$

where:

(6) βUI i,k=(1−Ri,k100) ∀ i ∈UI, ∀ k

The streams treated in each interceptor unit IU are sent to the process sink through separator $SUI_{i}$ Equation (7) shows the overall balance in this unit. The contaminant concentration of every stream treated in each intercept is equal to the contaminant concentration of the segregate stream, as seen in Equation (8).

(7) $$FIU_{i}^{out}=\underset{d\;\in UD}{\sum }FID_{i,d}+FIM_{i}\;\forall i\in IU$$

(8) $$xSIU_{i,k}^{out}=xIU_{i,k}^{out}\;\forall \;i\;\in UI,\;\forall \;k$$

The streams from the splitter treatment units are directed to the MD mixer demand unit, and the output stream is directed to the demand unit. The overall material balance for the mixer demand unit is given by Equation (9) and the mass balance for each contaminant *j* by Equation (10).

(9) $$FUD^{inlet}{}_{d}=F_{freshwater}+\underset{i\;\in UI}{\sum }FID_{i,d}+\underset{r\;\in US}{\sum }FSD_{r,d}\;\forall d\;\in UD$$

(10) $$FUD^{inlet}{}_{d}*xDU_{d,k}^{in}=\underset{i\;\in UI}{\sum }FID_{i,d}*xUI_{i,k}^{out}+\underset{r\;\in US}{\sum }FSD_{r,d}*xUS_{r,k}^{out}\;\forall \;d\in UD,\;\forall \;k$$

In the sink unit, flowrate and composition of specific chemical compounds, such as process constraint $xUD_{d,j}^{in,min}\leq \;xUD_{d,j}^{in,max}$, are included. On the other hand, the freshwater balance is raised through Equations (11) and (12).

(11) $$F_{fw}=\underset{d\in UD}{\sum }F_{fw,d}$$

(12) $$\underset{d\in UD}{\sum }F_{fw,d}\leq Pinch$$

Finally, a final mixer is included that receives the streams from the intercepted units that do not comply with the sink restrictions. The overall material balance for the final mixer is given by Equation (13) and the mass balance equation for each contaminant k by Equation (14).

(13) $$FMF^{out}=\underset{i\in IU}{\sum }FIM_{i}$$

(14) $$FMF^{out}*x_{k}^{out}=\underset{i\in IU}{\sum }FIM_{i}*xIU_{i,k}^{out},\forall \;k$$

The objective function is formulated to minimize the total network cost consisting of the cost of freshwater, the cost of investment on treatment units, and the operating cost for the treatment units (it is considered as one-third of the investment), as shown in Equation (15) [26].

(15) $$Min\;Z=AR*\sum_{i\in IU}CIU*{\left(FUI_{i}^{out}\right)}^{\alpha }+\frac{1}{3}\;\sum_{i\in IU}CIU*{\left(FUI_{i}^{out}\right)}^{\alpha }+H*F_{fw\;}*CU_{fw}$$

Finally, the proposed optimization model is an NLP type, taking into account the type of variables, the nature of the restrictions, and the objective function. Its solution can be carried out using computational tools such as LINDO, EMSO, MATLAB, MINOPT, and GAMS, among others.

## 3. Results

As a case study, the shrimp shell wastes processing scheme was chosen for the production of chitosan from the shrimp exoskeleton.

### 3.1. Process Modeling and Simulation

The flow diagram was constructed from information reported in the literature, 6600 kg/h of shell wastes was taken as the basis of calculation according to the availability of the raw material; the scheme was simulated with the help of the Aspen Plus simulator in a stable state, considering 3 main functional blocks, which are explained below. The thermodynamic model for property estimations was the electrolyte non-random two-liquid (eNRTL).

**Figure 3:**
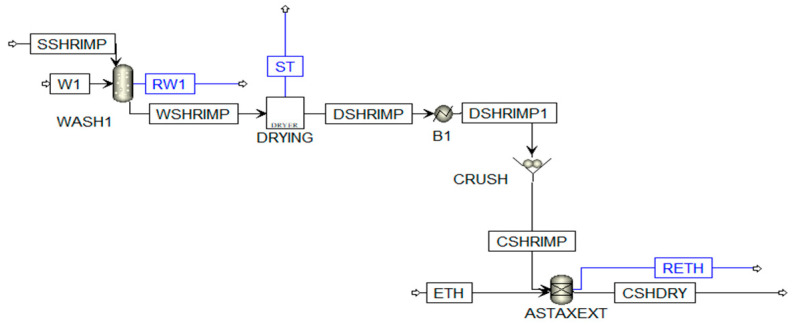
Shell waste pretreatment. Adapted from Meramo-Hurtado et al. [28].

The first stage is pretreatment (see Figure 3). The raw material composition (SSHRIMP) was modeled considering amino acids, fatty acids, carbonates, and pigment according to what was reported by Gómez-Ríos et al. [27]. This stage includes physical operations such as washing to remove impurities using a water/raw material ratio of 10/1, size reduction up to 5 mm to homogenize the sample using a crusher with a cut off size ratio of 6, and extraction of organic compounds (astaxanthin) using ethanol–water 85% *w*/*w*. The CHSDRY stream is directed to the demineralization stage, where minerals like calcium carbonate are removed by adding a solution of de-hydrochloric acid 5% *w*/*w*; reactions Equations (16)–(19) were modeled through a conversion reactor (RStoic).

(16) $${\mathrm{CaCO}}_{3}+2\mathrm{HCL}\to {\;\mathrm{CaCl}}_{2}+H_{2}O+{\mathrm{CO}}_{2}$$

(17) $${\mathrm{Na}}_{2}{\mathrm{CO}}_{3}+2\mathrm{HCL}\to \;2\mathrm{NaCl}+H_{2}O+{\mathrm{CO}}_{2}$$

(18) $${\mathrm{MgCO}}_{3}+2\mathrm{HCL}\to {\;\mathrm{MgCl}}_{2}+H_{2}O+{\mathrm{CO}}_{2}$$

(19) $${\mathrm{Ca}}_{3}{\left({\mathrm{PO}}_{4}\right)}_{2}+6\mathrm{HCL}\to \;3{\mathrm{CaCl}}_{2}+2H_{3}{\mathrm{PO}}_{4}$$

Next, the CHD1 stream was subjected to deproteinization using a sodium hydroxide 2% *w*/*w* solution; reactions Equations (20)–(24) were simulated in a conversion reactor at 71 °C, obtaining the chitin as the main product.

(20) $$C_{6}H_{12}N_{2}O_{3}+2\mathrm{NaOH}\to \;2C_{3}H_{6}{\mathrm{NNaO}}_{2}+H_{2}O$$

(21) $$C_{10}H_{16}N_{2}O_{7}+2\mathrm{NaOH}\to 2C_{5}H_{8}{\mathrm{NNaO}}_{4}+H_{2}O$$

(22) $$C_{18}H_{20}N_{2}O_{3}+2\mathrm{NaOH}\to 2C_{9}H_{10}{\mathrm{NNaO}}_{4}+H_{2}O$$

(23) $$C_{10}H_{20}N_{2}O_{3}S_{2}+2\mathrm{NaOH}\to 2C_{5}H_{10}{\mathrm{NNaO}}_{2}S\;+H_{2}O$$

(24) $$C_{12}H_{26}N_{4}O_{3}+2\mathrm{NaOH}\to 2C_{6}H_{13}N_{2}{\mathrm{NaO}}_{2}+H_{2}O$$

(25) $$C_{8}H_{15}{\mathrm{NO}}_{6}+\mathrm{NaOH}\to C_{6}H_{13}{\mathrm{NO}}_{5}+C_{2}H_{3}{\mathrm{NaO}}_{2}$$

Finally, chitin was reacted with 50% *w*/*w* sodium hydroxide at high temperature, as shown in reaction 25, and chitosan was obtained, quantifying 1400 kg of chitosan/6602 kg of processed shrimp shell wastes.

The described scheme presents a high freshwater requirement (w1–w7) quantified in 387 L/kg of chitosan. Water availability through the waste streams was observed, which justifies the application of the integration strategy mass.

### 3.2. Targeting of Minimal Freshwater Usage

**Figure 4:**
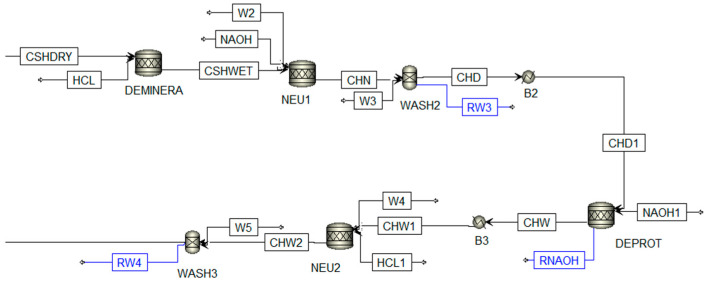
Demineralization and deproteinization reactions. Adapted from Meramo-Hurtado et al. [28].

**Figure 5:**
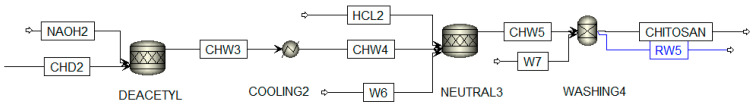
Deacetylation of chitin adapted from Meramo-Hurtado et al. [28].

The minimum amount determination of freshwater was carried out following the methodology in five sequential stages as described below:

In the first stage, the streams named source (r) with their respective flow and composition were identified. According to Figure 3, Figure 4 and Figure 5, the selected source streams were RW1, RW3, RW4, and RW5, which are at environmental conditions and have water availability. The sink (d) units are identified: WASH1, NEU1, WASH2, NEU2, WASH3, NEU3, and WASH 4, considering the required flow. In this case, the neutralization units were taken in the analysis as a single sink. The compounds called pollutants k were selected with the sink and source information, which present restrictions in terms of composition for the sink. For this process diagram, the selected pollutants were organic compounds (like astaxanthin, methyl palmitate, and ethanol) and salts resulting from neutralization, as shown in Table 1.

The second stage corresponds to identifying the limiting pollutant for each sink and determining the most likely source prioritization sequence for each sink. For this, the relationship proposed in Equation (16) was used, using the maximum concentration of each pollutant in the source (*C_organic_* = 1100 ppm and *C_NaCl_* = 350 ppm).

(26) $$\frac{Z_{*k,skj}}{C_{*k,sr\_max}}=\frac{Z_{K,rj}-C_{kFW}}{C_{k,rmax}-C_{kFW}}$$

Table 2 shows the results obtained where lower ratios <1 were evidenced, indicating a limitation of the sink by pollutants. In this case, the pollutant with the lowest ratio for each sink was chosen, being the cascade pollutant organic d1 and d3 and NaCl contaminant cascade for d2 and d4.

The total flows of the sinks were accounted for. In other words, for the case study, if the total flow d1 + d3 (331,239 kg /h) is higher than the total flow d2 + d4 (206,101 kg/h), then the pinch analysis will start on the organic cascade pollutant. Additionally, the source order was identified to fill the sinks according to the pollutant concentration. In this case, it was r1–r2–r3–r4 for the two pollutants.

At this stage, the pollutant sequence can also be defined for pinch analysis, taking into account the total flows of the sinks. In other words, for the case study, if the total flow d1 + d3 (331,239 kg /h) is higher than the total flow d2 + d4 (206,101 kg/h), then the pinch analysis will start on the organic cascade pollutant. Additionally, the source order was identified to fill the sinks according to the pollutant concentration. In this case, it was r1–r2–r3–r4 for the two pollutants. Finally, the required freshwater flow calculation was performed for each pollutant, applying the material recovery pinch diagram technique, where the compound curves (CC) for the source and sink are drawn in a plot with axes impurity load vs. cumulative flow rate. Then, the Source CC was shifted until it was on the right side of the Sink CC [25].

**Figure 6:**
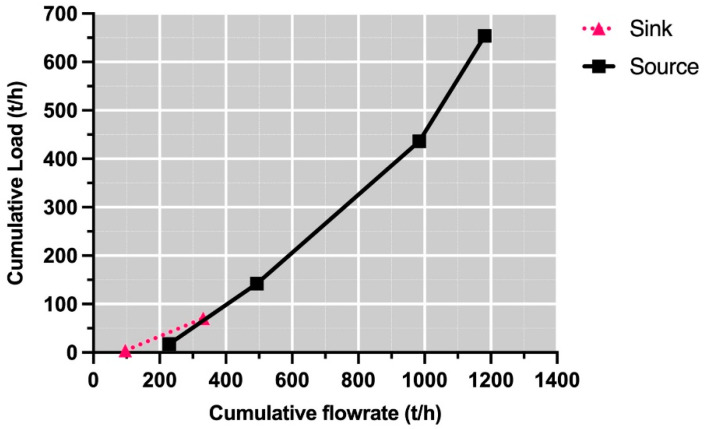
Composite curve for organic cascade.

As indicated, the calculation begins for the organic pollutant cascade, since it is the one that requires the highest flow. In Figure 6, the freshwater target can be identified as 230 t/h.

**Figure 7:**
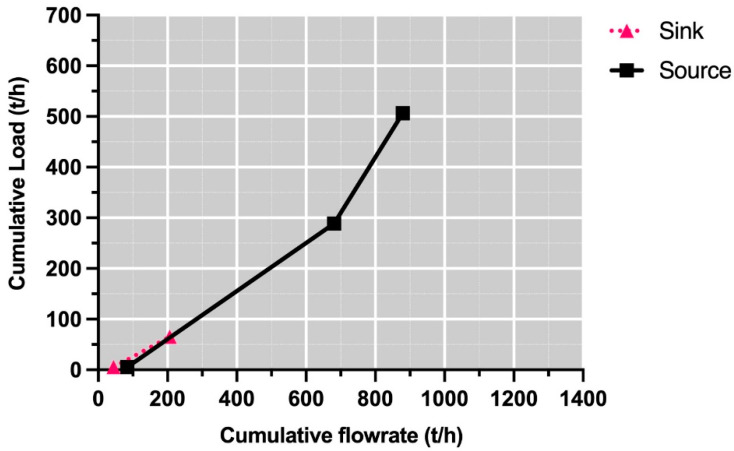
Composite curve for NaCl.

With respect to NaCl (see Figure 7), a freshwater requirement of approximately 90 t/h can be identified. In this way, 320 t/h can be established as the minimum freshwater consumption target, achieving a reduction of 40% with respect to the initial requirement.

In order to build the water network with reuse/recycle and regeneration strategies, the water network design is proposed using the mathematical programming approach to take even more advantage of the flow available in the source units [29]. It is based on the optimization of a superstructure, presented in Figure 2. For the case study, reverse osmosis is proposed as a regeneration unit due to the removal effectiveness of the compounds selected as pollutants [18,30]. The investment cost (COI) for the treatment unit (reverse osmosis) is shown in Equation (15). The latter was taken according to what was reported by Ahmetović et al. [26] and updated with the index cost to 2019.

COI = 3311.13 m (t/h)(27)

where m represents mass flowrate. The unit costs for the freshwater were taken from 1 dollar/m^3^, considering the Colombian regulations for water consumption in industry.

### 3.3. Synthesis of the Water Network

**Figure 8:**
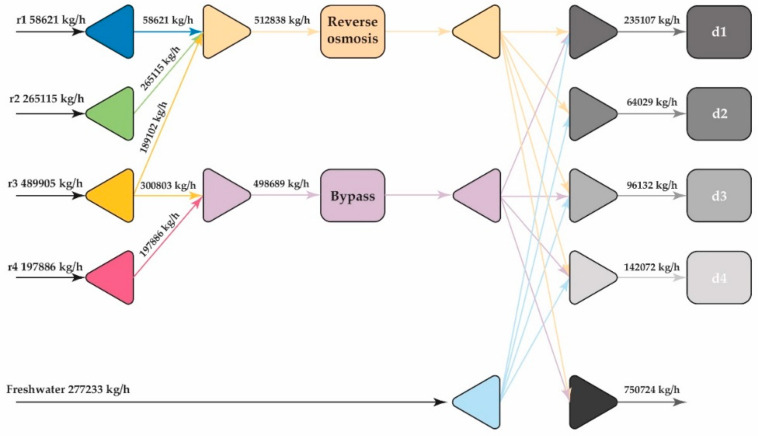
Optimal design for the water network.

The superstructure shown in Figure 3 was solved with the help of GAMS, taking into account Equations (1)–(15) that represent the mathematical model. The solution was found after 915 iterations at node 915, with an execution time of 7.3 s, obtaining a distribution of the stream as shown in Figure 8.

Figure 8 shows that the optimal design of the water network proposes a maximum theoretical reduction in freshwater consumption of 48%, integrating reverse osmosis as an intercept unit with removal percentages of 90% for the compounds identified as pollutants. The freshwater supply is distributed in all the sink units, directing about 49% to sink 1 and 22% to sink 4; this is due to its flow requirements and restrictions regarding organic components and NaCl. On the other hand, high water availability is observed in the final mixer, which could be used in other activities within the case study industry.

On the other hand, the mathematical model used for the mass integration of the present case study shows a total annual cost of US $5.5 million. The applied methodology that involves pinch analysis and mathematical programming gives comparable results with works reported in the literature. Balla et al. [31] analyze the sugar manufacturing process, using the technique from water cascade analysis (pinch analysis), showing a decrease in freshwater consumption of 43%, and 67% in wastewater discharge. Lee et al. [23] develop a mathematical model applied to an existing water network in a pulp and paper mill for direct reuse/recycling strategies and regeneration schemes using mono- and multi-objective optimization to minimize freshwater consumption and reasonable payback.

## 4. Conclusions

The methodology proposed in this paper allows identifying the minimum freshwater consumption through the pinch approach, taking into account the limitation in the sink for multiple pollutants. It was also possible to obtain a prioritization strategy for source and sink, taking into account the concept of reuse. Next, using mathematical programming techniques, it was possible to establish an optimal design for the water network, minimizing the total annualized cost of the network. For the case study, it was possible to identify a consumption reduction of 40% using only reuse strategies and of 48% using reuse and regeneration strategies, which provides a more holistic vision for decision making.

## Figures and Tables

**Table 1:** Date case study.

Source	Flow Rate kg/h	$C_{organic}\left(ppm\right)$	$C_{NaCl}\left(ppm\right)$
r1	58,621	300	10
r2	265,115	470	200
r3	489,905	600	270
r4	197,886	1100	350
Sink	Flowrate kg/h	$Z_{organic}\left(ppm\right)$	$Z_{NaCl}\left(ppm\right)$
d1	235,107	50	20
d2	64,029	20	10
d3	96,132	40	15
d4	142,072	25	10

**Table 2:** Source prioritization sequence/contaminant cascade of each sink.

	$Z_{*organic}$ $/C_{*organic,\_max}$	$Z_{*NaCl}$ $/C_{*Nacl\_max}$	Minimum Ratio	Cascade
d1	0.045	0.057	0.045	Organic
d2	0.018	0.017	0.017	NaCl
d3	0.037	0.043	0.037	Organic
d4	0.022	0.017	0.017	NaCl

## Data Availability

The data that support the findings of this study are available in the open literature, particularly the work of Meramo-Hurtado et al. [28].

## Abbreviations

**Sets and Indices**	
k	Contaminant
r	Unit source
i	Unit intercept
IU	Set of intercept unit
SU	Set of source unit
**Parameters**	
AR	Annualized factor
α	Cost exponent for intercept unit
H	Hours of plant operation per year
CIU	Investment cost of treatment unit
$CU_{fw}$	Cost of freshwater
$FSU_{r}^{out}$	mass flowrate of outlet stream from source unit r
$xUS_{r,k}^{out}$	concentration of contaminant k in outlet stream from source unit r
$R_{i,k}$	% removal of contaminant k in intercept unit t
$xUD_{d,j}^{in,max}$	maximum concentration of contaminant k in inlet stream into demand unit d
**Continuous Variables**	
$FSI_{r,i}\;$	mass flowrate of stream from unit source r to intercept unit I
$FUI_{i}^{inlet}$	mass flowrate of inlet stream intercept unit I
$FUI_{i}^{out}$	mass flowrate of outlet stream from intercept unit I
$FSI_{r,i}$	mass flowrate of water stream from source unit r to treatment unit i
$xIU_{i,k}^{inlet}$	concentration of contaminant k in inlet stream into intercept unit
$xUI_{i,k}^{out}$	concentration of contaminant k in outlet stream from intercept unit I
$FID_{i,d}$	mass flowrate of stream from unit intercept i to demand unit d
$FIM_{i}$	mass flowrate of water stream from intercept unit i to final mixer
$xSIU_{i,k}^{out}$	concentration of contaminant k in outlet stream from splitter treatment unit I
$FUD^{inlet}{}_{d}$	mass flowrate of inlet water stream in deposit unit d
$F_{freshwater}$	mass flowrate of freshwater
$FSD_{r,d}$	mass flowrate of water stream from source unit r to deposit unit d
$xSUD_{d,k}^{in}$	concentration of contaminant k in inlet stream into deposit unit d
$xUS_{r,k}^{out}$	concentration of contaminant k in outlet stream from source unit r
$xUD_{d,k}^{in,min}$	minimum concentration of contaminant k in inlet stream into demand unit d
